# Long fasting is effective in inhibiting physiological myocardial ^18^F-FDG uptake and for evaluating active lesions of cardiac sarcoidosis

**DOI:** 10.1186/2191-219X-4-1

**Published:** 2014-01-02

**Authors:** Miyako Morooka, Masao Moroi, Kimiichi Uno, Kimiteru Ito, Jin Wu, Takashi Nakagawa, Kazuo Kubota, Ryogo Minamimoto, Yoko Miyata, Momoko Okasaki, Osamu Okazaki, Yoshihito Yamada, Tetsuo Yamaguchi, Michiaki Hiroe

**Affiliations:** 1 Division of Nuclear Medicine, Department of Radiology, National Center for Global Health and Medicine, Shinjuku-ku, Tokyo 162-8655, Japan; 2 Department of Cardiology, National Center for Global Health and Medicine, 1-21-1 Toyama, Shinjuku-ku, Tokyo 162-8655, Japan; 3 Division of Cardiovascular Medicine, Toho University Ohashi Medical Center, 2-17-6 Ohashi, Meguro-ku, Tokyo 153-8515, Japan; 4 Gaien Higashi Clinic, 20 Samontyo, Shinjuku-ku, Tokyo 160-0017, Japan; 5 Department of Respiratory Medicine, JR Tokyo General Hospital, 2-1-3 Yoyogi, Shibuya-ku, Tokyo 151-0053, Japan

**Keywords:** Glucose utilization, Heparin loading, Myocardial cells, Inflammatory cells, Test preparation

## Abstract

**Background:**

F-fluorodeoxyglucose (FDG) positron emission tomography (PET) is a promising modality for detecting active lesions of cardiac sarcoidosis (CS). However, determining whether ^18^F-FDG uptake in the myocardium is physiological is challenging due to metabolic shift in myocardial cells. Although methods for inhibiting physiological myocardial ^18^F-FDG uptake have been proposed, no standard methods exist. This study therefore aimed to compare the effect of an 18-h fast (long fasting (LF)) with heparin loading plus a 12-h fast (HEP) before ^18^F-FDG PET scan.

**Methods:**

We analyzed the effects of LF and HEP on the inhibition of physiological myocardial ^18^F-FDG uptake in healthy subjects (18 in HEP and 19 in LF) and in patients with known or suspected CS (96 in HEP and 69 in LF). In CS, the lower uptake of ^18^F-FDG in the myocardium was evaluated. A visual four-point scale was used to assess myocardial ^18^F-FDG uptake in comparison with hepatic uptake (1 lower, 2 similar, 3 somewhat higher, 4 noticeably higher).

**Results:**

Myocardial ^18^F-FDG uptake was 1.68 ± 1.06 in LF and 3.17 ± 1.16 in HEP in healthy subjects (*p* < 0.0001), whereas it was 1.48 ± 0.99 in LF and 2.48 ± 1.33 in HEP in CS patients (*p* < 0.0001). Logistic regression and regression trees revealed the LF was the most effective in inhibiting myocardial ^18^F-FDG uptake. In addition, serum free fatty acid levels on intravenous ^18^F-FDG injection were a possible biomarker.

**Conclusions:**

LF is effective in inhibiting myocardial ^18^F-FDG uptake, and consequently, it could be useful for evaluating active lesions of CS in ^18^F-FDG PET images.

## Background

Detecting and managing cardiac sarcoidosis (CS) is challenging even for expert physicians. The Japanese Ministry of Health and Welfare has published guidelines for diagnosing CS [1], while The Joint Statement of the American Thoracic Society, the European Respiratory Society, and the World Association for Sarcoidosis and Other Granulomatous Disorders have proposed a definition of CS [2]. However, there is no convincing consensus on the optimal methods for disease detection, monitoring, and treatment.

^18^F-fluorodeoxyglucose (FDG) positron emission tomography (PET) is a promising modality for detecting ‘active’ lesions in various inflammatory cardiovascular diseases, including cardiac involvement of sarcoidosis, myocarditis, and vascular diseases such as Takayasu's arteritis, giant cell arteritis, and atherosclerosis [3-5]. However, assessing inflammatory cardiomyopathies using ^18^F-FDG PET can be challenging because the radiotracer accumulates in normal myocardium, a phenomenon known as physiological uptake; this is problematic because the myocardial uptake of ^18^F-FDG is heterogeneously based on metabolic shifts in myocardial cells [6-8].

Methods proposed for the inhibiting increased ^18^F-FDG uptake in myocardial physiological cells include heparin, long fasting, and dietary carbohydrate restriction before the scan [4,5,9-15]. These methods are believed to be associated with reduced blood insulin and increased circulating free fatty acid (FFA) levels; however, no study has determined which of these methods is the most appropriate. The aim of the present study was to assess the effects of an 18-h fast (long fasting (LF)) on inhibiting physiological myocardial ^18^F-FDG uptake compared with heparin loading plus a 12-h fast (HEP) in healthy subjects and patients with known or suspected CS.

## Methods

### Subjects

**Figure 1:**
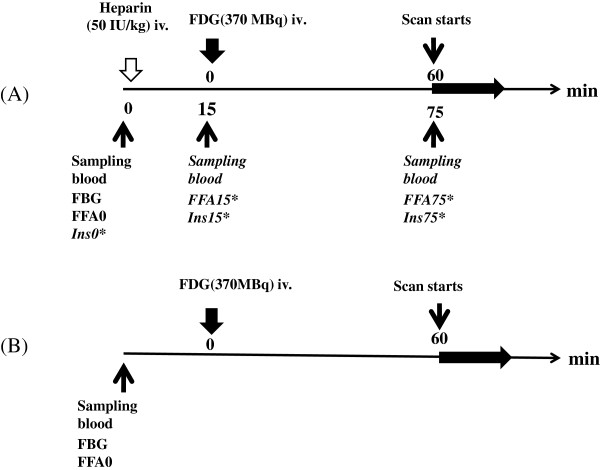
**Protocol of **^**18**^**F-FDG PET/CT with (A) and without (B) heparin loading.** Heparin (50 IU/kg) was intravenously injected 15 min before the injection of ^18^F-FDG, and the can commenced 60 min after ^18^F-FDG injection. Venous blood sampling was performed 15 min before ^18^F-FDG injection and 15 and 75 min after heparin injection in health subjects with heparin loading plus a 12-h fast (HEP group). In the healthy subjects with no heparin loading plus fasting for a minimum of 16 h (LF group), venous blood sampling was performed 15 min before ^18^F-FDG injection.

Healthy subjects had no history of cardiac disease or risk factors, a body mass index (BMI) of <30, no diabetes mellitus, no history of illness, and normal electrocardiography findings. Eighteen healthy subjects underwent ^18^F-FDG PET with an intravenous injection of heparin (50 IU/kg) 15 min before ^18^F-FDG injection with fasting for 12 h before ^18^F-FDG PET scan (HEP group), and 19 subjects were not injected with heparin but they fasted for a minimum of 16 h (LF group) (Figure 1). FDG PET in normal volunteers of HEP group was performed from February 26, 2009 to October 27, 2010, and that of LF group was done from February 21, 2013 to July 18, 2013. In the 18 HEP subjects, blood samples for measuring plasma FFA levels were obtained immediately before (baseline (FFA0)) and 15 min after heparin injection (FFA15). In 9 of the 18 subjects, additional blood samples were obtained before starting the scan, and insulin levels were measured (FFA levels 75 min after heparin injection (FFA75), baseline insulin levels (Ins0), insulin levels 15 min after heparin injection (Ins15), insulin levels 75 min after heparin injection (Ins75)) (Figure 1).

In patients with known or suspected CS, ^18^F-FDG PET with HEP was performed between January 2009 to June 2012, and ^18^F-FDG PET with LF was performed between July 2012 and July 2013. Consequently, a total of 96 patients in the HEP group and 69 patients in the LF group were included in the study. Patients with histological (or clinical) diagnosis of extra-cardiac sarcoidosis, with or without cardiac symptoms, were enrolled in the study. Patients who had not been diagnosed with extra-cardiac sarcoidosis but were suspected of having the condition based on clinical data (e.g., a 55-year-old female with unexplained sustained second- or third-degree atrioventricular block or with sustained monomorphic ventricular tachycardia) were also included in this study [1,16]. The guidelines of the Japanese Society of Sarcoidosis and Other Granulomatous Disease are presented in Table 1. For patients in the HEP group, the blood samples at baseline were collected after a 12-h fast for measuring fasting blood glucose and FFA levels (i.e., FFA0), before unfractionated heparin (50 IU/kg) was injected (Figure 1). Fifteen minutes after heparin injection, ^18^F-FDG (370 MBq) was intravenously injected. For patients in the LF group, the blood samples at baseline were collected for measuring fasting blood glucose and FFA0 levels 15 min before intravenous injection of ^18^F-FDG (370 MBq).

**Table 1:** The Japanese Society of Sarcoidosis and Other Granulomatous Disorders' guidelines for CS diagnosis (2006)

**Diagnosis group**	**Guideline**
Histological diagnosis group	Cardiac sarcoidosis is confirmed when endomyocardial biopsy specimens demonstrate noncaseating epithelioid cell granulomas with a histological or clinical diagnosis of extracardiac sarcoidosis.
Clinical diagnosis group	Although endomyocardial biopsy specimens do not demonstrate noncaseating epithelioid cell granulomas, extracardiac sarcoidosis is histologically or clinically diagnosed and satisfies the following conditions and more than one in six basic diagnostic criteria.
	1. Two or more of the four major criteria are satisfied.
	2. One of the four major criteria and two or more of the five minor criteria are satisfied.
	Major criteria
	a. Advanced atrioventricular block
	b. Basal thinning of the interventricular septum
	c. Positive 67 gallium uptake in the heart
	d. Depressed ejection fraction of the left ventricle (<50%)
	Minor criteria
	a. Abnormal ECG findings: ventricular arrhythmias (ventricular tachycardia, multifocal or frequent PVCs), CRBBB, axis deviation or abnormal Q-wave
	b. Abnormal echocardiography: regional abnormal wall motion or morphological abnormality (ventricular aneurysm, wall thickening)
	c. Nuclear medicine: perfusion defect detected by 201 thallium or 99 mtechnetium myocardial scintigraphy
	d. Gadolinium-enhanced CMR imaging: delayed enhancement of the myocardium
	e. Endomyocardial biopsy: interstitial fibrosis or monocyte infiltration over moderate grade.

CMR, cardiac magnetic resonance; CRBBB, complete right bundle branch block; CS, cardiac sarcoidosis; ECG, electrocardiography; PVC, premature ventricular contraction.

The Review Board of the National Center for Global Health and Medicine approved the study protocol. In accordance with the Declaration of Helsinki, all patients provided written informed consent before enrollment in the study.

### ^18^F-FDG PET scan

PET-computed tomography (CT) (Biograph Siemens 16 and Siemens, Malvern, PA, USA and Discovery PET/CT 600 M, GE, Fairfield, CT, USA) scan of the heart was commenced with a 10-min emission scan/bed (one bed position covering a 16-cm field of view along *X*-*Y*-*Z* axes) 1 h after the intravenous injection of ^18^F-FDG. A whole-body PET-CT scan was subsequently performed from the vertex to the mid-thighs. Attenuation-corrected PET-CT images were reconstructed using the CT data, and the PET data were reconstructed using a combination of FORE and OSEM algorithms (four iterations, eight subsets; Siemens, Munich) and the 3D-OSEM algorithm (three iterations, 16 subsets; GE) using a Gaussian filter.

### Evaluation of myocardial ^18^F-FDG uptake

Two experienced cardiologists and two nuclear medicine specialists determined whether the increased ^18^F-FDG uptake in the myocardium was physiological or caused by active CS. These decisions were based on the findings of physiological normal images obtained from the healthy subjects, clinical data such as electrocardiographs, cardiac echo studies, ^201^Tl/^123^I β-methyl-*p*-iodophenyl-pentadecanoic acid (BMIPP) single-photon emission computed tomography image, and cardiac magnetic resonance imaging.

### Visually semi-quantitative analysis in healthy subjects or patients with known or suspected CS

**Figure 2:**
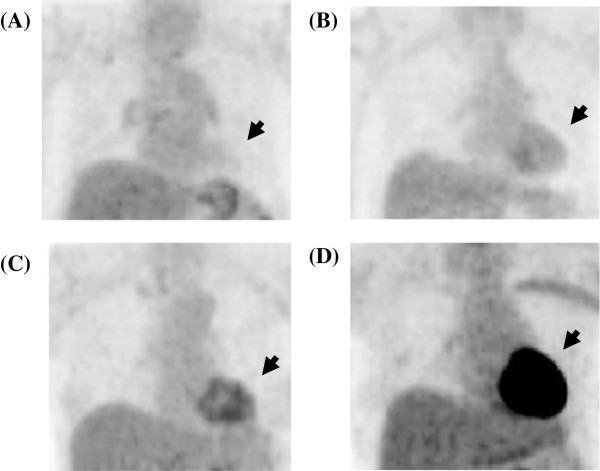
**Visual evaluation of myocardial **^**18**^**F-FDG uptake using a four-point scale.** Myocardial ^18^F-FDG uptake was lower than hepatic uptake (**A**, grade 1), similar to hepatic uptake (**B**, grade 2), somewhat higher than hepatic uptake (**C**, grade 3), and noticeably higher than hepatic uptake (**D**, grade 4). Grade 1 can be recognized as a complete inhibition of physiological uptake.

The ^18^sF-FDG PET images obtained from the healthy subjects and patients with known or suspected CS in the HEP and LF groups were visually assessed and analyzed using a modified four-point scale (grade 1, myocardial ^18^F-FDG uptake lower than hepatic uptake; grade 2, myocardial ^18^F-FDG uptake similar to hepatic uptake; grade 3, myocardial ^18^F-FDG uptake somewhat higher than hepatic uptake; and grade 4, myocardial ^18^F-FDG uptake noticeably higher than hepatic uptake) [17]. Two experienced nuclear medicine physicians who were unaware of the clinical findings or previous imaging results independently performed the assessment and analysis. Grading of the physiological uptake in ^18^F-FDG images is shown in Figure 2, where grade 1 can be recognized as a complete inhibition of physiological uptake.

### Quantitative analysis using standardized uptake value in healthy subjects

In healthy subjects, a region of interest (ROI) was placed on the maximal accumulation in the basal area, and the standardized uptake value (SUV_max_) was measured. In patients with known or suspected CS, the SUV_max_ was not measured because an SUV in the basal area may be an active lesion of sarcoidosis; thus, a definite ROI could not be placed to measure the SUV_max_ for physiological uptake.

### Statistical analysis

Data are expressed as mean ± standard deviation (SD) of continuous variables, and the Pearson correlation coefficient and linear regression analysis were used to analyze the relationship between two variables. The Student's *t* test was used to compare variables in healthy subjects and patients with known or suspected CS. Logistic regression analysis was used to determine significant predictors of inhibiting physiological myocardial ^18^F-FDG uptake. Finally, regression tree analysis was used to determine the most significant factor in the clinical setting to inhibit physiological myocardial ^18^F-FDG uptake.

Statistical analyses were performed using SPSS software (SPSS Statistics for Windows, version 20.0, IBM Corp., Armonk, NY, USA) for the Student's *t* test, Pearson correlation coefficient, and linear regression analysis. JMP software (version 10, SAS Institute Inc., Cary, NC, USA) was used for the logistic regression and regression tree analysis. Statistical significance was defined as *p* < 0.05.

## Results

### Differences between HEP and LF and factors for inhibiting physiological myocardial ^18^F-FDG uptake in healthy subjects

**Figure 3:**
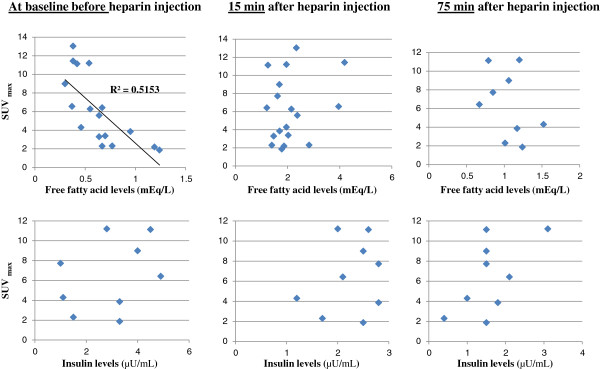
**Association between SUV**_**max**_**, FFA levels, Ins0, Ins15, and Ins75 in healthy subjects.** The upper three graphs show the relationship between the SUV_max_ and FFA levels. A significant correlation was observed between the SUV_max_ and baseline FFA levels immediately before heparin injection (*r* = −0.7178, *p* < 0.05). The bottom three graphs show the relationship between the SUV_max_ and insulin levels; no correlation was observed.

The characteristics and blood sampling data of the healthy subjects in the HEP and LF groups are summarized in Tables 1 and 2, respectively. The fasting blood glucose levels were <110 mg/dL; there was no difference with regard to the glucose levels between the HEP and LF groups. The plasma FFA15 levels increased twofold to threefold in comparison with the FFA0 levels. However, the plasma FFA75 levels decreased at the commencement of scanning (numbers 10 to 18 in Table 2). In many cases, the Ins15 levels decreased in comparison with the Ins0 levels, and the Ins75 levels further decreased in comparison with the Ins15 levels. A correlation between FFA0 levels and SUV_max_ was observed in subjects in the HEP group (*r* = 0.72, *p* < 0.05; Figure 3). In contrast, there was no correlation between SUV_max_ and FFA15, FFA75, Ins0, Ins15, or Ins75 levels (Figure 3). An increase in baseline serum FFA levels may inhibit physiological myocardial ^18^F-FDG uptake. Visual semi-quantitative analysis revealed that only 2 subjects (11.1%) of the 18 subjects in the HEP group had grade 1 physiological myocardial ^18^F-FDG uptake.

**Table 2:** Healthy volunteers with heparin loading method

**No.**	**Age (years)**	**Gender**	**BMI**	**Heparin (IU)**	**FBG (mg/dL)**	**FFA0**	**FFA15**	**FFA75**	**Ins0**	**Ins15**	**Ins75**	**Phy U**	**SUV**_**max**_	**Polar map pattern**
							**(mEq/L)**			**(μU/mL)**				
1	33	Male	23.4	3,500	99	0.77	2.83					2	2.31	Diffuse
2	34	Male	23.8	4,000	94	0.38	2.34					4	13.04	Basal lateral
3	36	Male	21.6	3,500	90	1.19	1.87					1	2.2	
4	38	Female	21.8	2,500	108	0.37	3.96					4	6.56	Basal lateral
5	48	Male	23.4	4,000	104	0.55	2.15					4	6.28	Basal lateral
6	49	Male	21.4	3,500	100	0.38	4.19					4	11.43	Basal lateral
7	58	Male	21.4	3,000	99	0.7	2.03					2	3.39	Basal lateral
8	60	Male	25.3	3,300	106	0.64	2.38					4	5.58	Basal lateral
9	76	Female	23.2	3,000	96	0.64	1.47					2	3.3	Diffuse
10	29	Male	20.8	3,000	82	0.54	1.96	1.2	2.8	2	3.1	4	11.2	Basal lateral
11	32	Female	19.5	3,000	90	0.67	1.21	0.67	4.9	2.1	2.1	4	6.42	Diffuse
12	40	Female	19.5	2,500	102	0.3	1.69	1.06	4	2.5	1.5	4	8.99	Diffuse
13	45	Male	21.53	3,300	102	0.46	1.96	1.52	1.1	1.2	1	3	4.29	Basal lateral
14	47	Female	20	3,000	92	1.24	1.78	1.24	3.3	2.5	1.5	1	1.87	
15	52	Male	23.9	4,000	108	0.42	1.25	0.79	4.5	2.6	1.5	4	11.13	Basal lateral
16	54	Female	22	3,000	109	0.67	1.4	1.01	1.5	1.7	0.4	2	2.29	Diffuse
17	55	Female	17.9	2,500	93	0.95	1.7	1.17	3.3	2.8	1.8	4	3.86	Basal lateral
18	62	Female	23.1	3,000	110	0.69	1.62	0.85	1	2.8	1.5	4	7.72	Basal lateral

BMI, body mass index; FBG, fasting blood glucose; FFA0, serum free fatty acid concentration at baseline (normal range 0.10 to 0.81 mEq/L); FFA15, serum free fatty acid concentration 15 min after heparin injection; FFA75, serum free fatty acid concentration 75 min after heparin injection; Ins0, serum insulin concentration at baseline; Ins15, serum insulin concentration 15 min after heparin injection; Ins75, serum insulin concentration 75 min after heparin injection; Phy U, physiological uptake (grading scale) 1 to 4; SUVmax, maximal standard uptake value.

In contrast, there was no linear correlation between the FFA0 levels and SUV_max_ in the subjects in the LF group (Table 3). However, 12 (63.2%) of the 18 subjects in the LF group presented with grade 1 physiological myocardial ^18^F-FDG uptake or complete inhibition of the uptake.

**Table 3:** Healthy volunteers with 18-hour fasting

**No.**	**Age (years)**	**Gender**	**BMI**	**Fasting duration (h)**	**FBG (mg/dL)**	**FFA0 (mEq/L)**	**Phy U**	**SUV**_**max**_	**Polar map pattern**
1	68	Male	27.4	18	86	0.56	1	3.54	
2	64	Female	22.3	18	90	0.5	1	3.06	
3	56	Male	22.5	16	87	0.63	3	7.23	Basal lateral
4	42	Male	27.1	23	88	0.24	4	20.35	Diffuse
5	64	Female	27.9	18	95	0.73	1	3.05	
6	66	Male	21.7	17	74	1.31	1	2.71	
7	39	Male	23.4	18	88	0.58	1	2.78	
8	41	Male	27.4	18	92	0.52	2	5.05	Basal lateral
9	72	Female	19.8	18	100	0.41	2	3.3	Basal lateral
10	44	Female	24.2	18	78	0.72	4	6.88	Diffuse
11	68	Female	22.2	18	77	0.96	1	2.13	
12	41	Female	19.0	18	75	0.273	2	3.62	Basal lateral
13	52	Male	28.6	18	78	0.539	3	5.9	Basal lateral
14	50	Female	23.3	22	78	0.725	1	1.84	
15	40	Male	29.0	18.5	107	0.649	1	1.61	
16	44	Male	21.8	18.5	68	0.731	1	1.55	
17	63	Male	19.7	18	73	0.594	1	1.31	
18	47	Male	25.4	18	79	0.872	1	1.48	
19	55	Male	30.0	18	104	0.547	1	1.83	

BMI, body mass index; FBG, fasting blood glucose; FFA0, free fatty acid at baseline (normal range; 0.10 to 0.81 mEq/L); Phy U, physiological uptake (grading scale) 1 to 4; SUVmax, maximal standard uptake value.

The physiological myocardial ^18^F-FDG uptake was lower in subjects in the LF group compared with the HEP group (Table 4). There were significant differences in physiological myocardial ^18^F-FDG uptake (3.17 ± 1.16 in HEP, 1.68 ± 1.06 in LF, *p <* 0.0001), fasting blood glucose levels (99.11 ± 7.84 in HEP, 85.11 ± 10.97 in LF, *p* < 0.0001), and BMI (21.86 ± 1.88 in HEP, 24.94 ± 3.28 in LF, *p* = 0.019) between subjects in the HEP and LF groups. There were no significant differences in age, gender, FFA0 levels, and SUV_max_ between the groups.

**Table 4:** Comparison of 18-h fasting with heparin loading plus 12-h fasting in healthy subjects

	**HEP**		**LF**		***p *value**
	***N***	**Average ± SD**	***N***	**Average ± SD**	
Age (year)	18	47.11 ± 12.53	19	53.47 ± 11.34	0.11
Gender	18	M/F = 10:8	19	M/F = 12:7	0.64
FBG	18	99.11 ± 7.84	19	85.11 ± 10.97	<0.0001***
FFA	18	0.64 ± 0.27	19	0.64 ± 0.24	0.94
BMI	18	21.86 ± 1.88	19	24.94 ± 3.28	0.019*
Phy U	18	3.17 ± 1.16	19	1.68 ± 1.06	<0.0001***
SUV_max_	18	6.21 ± 3.64	19	4.17 ± 4.31	0.13

HEP, heparin loading plus 12-h fasting; LF; 16-h or more fasting; FBG, fasting blood glucose (mg/dL); FFA, plasma free fatty acids (mEq/L); BMI, body mass index; Phy U, physiological uptake grades 1 to 4; *p < 0.05, ***p < 0.001.

**Figure 4:**
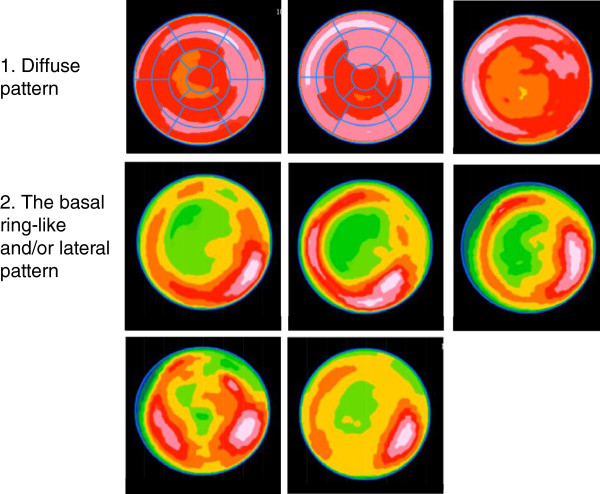
**Polar maps of healthy volunteers with myocardial FDG uptake.** There were two patterns of myocardial FDG uptake (grade 2, 3, or 4) in healthy volunteers when they had preparation with 12-h fast plus heparin injection (HEP) or long fast (16 h or more, LF). The polar map patterns include (1) a diffuse uptake pattern and (2) a basal ring-like and/or lateral uptake pattern.

There were two polar map patterns of location of the physiological myocardial uptake (grade 2, 3, or 4) in the left ventricular wall from the data of healthy subjects as shown in Figure 4: (1) a diffuse uptake and (2) basal ring-like and/or lateral uptake. Healthy subjects of the HEP group revealed diffuse uptake pattern in 31% (5/16) and basal ring-like and/or lateral uptake pattern in 69% (11/16, Table 2), whereas those of the LF group did diffuse uptake pattern in 29% (2/7) and basal ring-like and/or lateral uptake pattern in 71% (5/7, Table 3). Based on the polar map patterns observed in healthy subjects, we considered a diffuse uptake or basal ring-like and/or lateral uptake to be physiological in patients with suspected or known cardiac sarcoidosis.

Regression trees in all healthy subjects (*n* = 37) showed that the most significant factor that discriminated between grade 1 physiological myocardial ^18^F-FDG uptake and grade 2, 3, or 4 was ‘LF’. In subjects in the LF group, the most significant discriminatory factor for grade 1 physiological myocardial ^18^F-FDG uptake and grades 2, 3, or 4 was ‘FFA0’ ≥ 0.547 compared with ‘FFA0’ < 0.547.

### Differences between HEP and LF and factors for inhibiting physiological myocardial ^18^F-FDG uptake in patients with known or suspected CS

Patient characteristics and blood sampling data for subjects in the HEP and LF groups are summarized in Table 5. Ninety-six and 69 ^18^F-FDG PET scans were performed in patients with HEP and LF, respectively. The mean fasting time in the LF group was 20 h (range, 18 to 26 h). The differences in physiological uptake (2.46 ± 1.33 in HEP, 1.48 ± 0.99 in LF; *p <* 0.0001), fasting blood glucose levels (99.68 ± 14.12 in HEP, 92.80 ± 13.35 in LF, *p* = 0.002), and FFA0 levels (0.81 ± 0.39 in HEP, 1.02 ± 0.41 in LF, *p* = 0.001) were statistically significant. There were no significant differences in age, gender, BMI, and use of steroid therapy between the groups.

**Table 5:** Comparison between HEP and LF in patients with known or suspected cardiac sarcoidosis

	**HEP**		**LF**		***p *value**
	***N***	**Average ± SD**	***N***	**Average ± SD**	
Age (years)	96	57.19 ± 13.88	69	56.94 ± 13.16	0.91
Gender	96	M/F = 37:59	69	M/F = 17:52	0.06
FBG	96	99.68 ± 14.12	69	92.80 ± 13.35	0.002**
FFA	96	0.81 ± 0.39	69	1.02 ± 0.41	0.001**
BMI	96	22.37 ± 3.39	69	21.83 ± 3.08	0.28
Phy U	96	2.46 ± 1.33	69	1.48 ± 0.99	<0.0001***
Steroid use	96	19	69	14	0.98

HEP, heparin loading plus 12-h fasting; LF, 16-h or more fasting; FBG, fasting blood glucose (mg/dL); FFA, plasma free fatty acids (mEq/L); BMI, body mass index; Phy U, physiological uptake grades 1 to 4; ** p < 0.01; *** p < 0.001.

**Figure 5:**
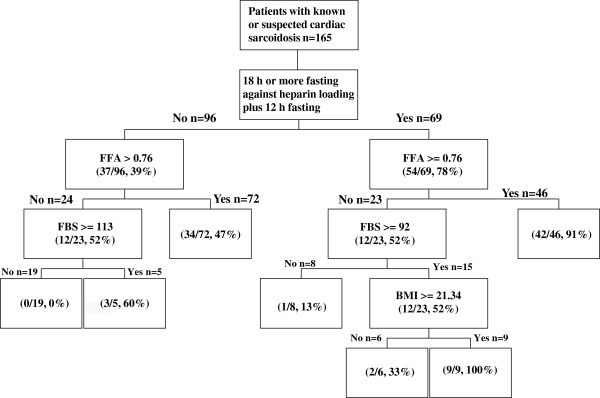
**Regression trees determining the factors that inhibit physiological myocardial **^**18**^**F-FDG uptake and their respective cutoffs.** The numbers in parentheses indicate the number and percentage of patients with grade 1 physiological myocardial ^18^F-FDG uptake.

Logistic regression analysis revealed that LF was the most important determinant of physiological uptake inhibition (Table 6). Classification and regression trees revealed that the most significant factor to determine between grade 1 physiological uptake and grades 2, 3, and 4 in all 165 patients was LF (Figure 5). In patients with LF, FFA0 ≥ 0.76 was a significant determinant of the inhibition of physiological uptake. When a patient with LF had FFA0 ≥ 0.76, the probability that physiological uptake would be inhibited was approximately 91%.

**Table 6:** Multivariate predictors of physiological uptake grade 1 in patients with known or suspected cardiac sarcoidosis

**Predictors**	**Odds ratio**	**95% Confidence interval**		***p *value**
		**Lower limit**	**Upper limit**	
Age (per 1-year increase)	0.994	0.968	1.021	0.677
Female	1.624	0.840	3.940	0.130
BMI (per 1-kg/m^2^ increase)	1.090	0.976	1.224	0.133
FBG (per 1-mg/dL increase)	0.992	0.966	1.017	0.517
FFA0 (per 1-mEg/L increase)	2.415	0.914	7.075	0.089
LF (against HEP)	4.708	2.268	10.172	<0.0001***

BMI, body mass index; FBG, fasting blood glucose; FFA0, baseline plasma free fatty acid levels; LF, 16-h or more fasting before scan; HEP; heparin loading plus 12-h fasting; *** p < 0.001.

## Discussion

Myocardial uptake is heterogeneous, and the glucose loading method is the standard protocol for detecting viability using ^18^F-FDG PET [18-20]. On the other hand, other methods attempt to detect inflammatory lesions in CS. These methods include the LF method, which involves a long fasting period before intravenous ^18^F-FDG injection [11-13], the consumption of a fatty meal [14,15], and the HEP method [4,5,9,10]. After heparin injection, plasma FFA levels acutely increase [9], reducing glucose consumption in the normal myocardium, and consequently this highlights, the FDG uptake in inflammatory lesions. It is believed that these methods result in high FFA levels, which inhibit physiological myocardial uptake.

In the present study, we compared the physiological uptake using the LF and HEP methods. We performed cardiac ^18^F-FDG PET using the LF and HEP methods in healthy subjects, and we observed that HEP did not reduce the overall physiological uptake compared with LF. Using the LF method, the physiological uptake was inhibited more efficiently in subjects with higher plasma FFA levels.

SUV_max_ was higher in patients in the HEP group than in the LF group, and it was inversely correlated with the FFA0 levels. Boden reported chronic increases in the FFA levels; when elevated FFA levels are chronically maintained, they cause peripheral (muscle) insulin resistance, inhibiting insulin-stimulated glucose uptake and glycogen synthesis [21-23]. FFA0 level reflects the chronic state in an individual. When the FFA0 level is high, physiological uptake of glucose in the myocardium is reduced. In this study, we used a single injection of heparin; thus, the FFA75 levels decreased after an initial increase in the FFA15 levels. That is a reason why physiological FDG uptake was not inhibited enough in HEP. Persistent FFA levels were inadequate in using a single 50 IU/kg dose. A longer continuous infusion of heparin may have been more successful. For another reason, there may have been effect of different diets, because the study subjects were not on a special diet (low carbohydrates and high fat). The low-carbohydrate and high-fat meal may help ensure an adequately high FFA0 [15].

In all patients with known or suspected CS, LF was the most important predictor as per logistic regression and regression trees. For patients in the LF group, the FFA0 levels were the most important factor, and the cutoff value of FFA0 levels was 0.76. LF is therefore the most important factor for inhibiting physiological uptake, and when FFA0 levels are more than 0.76 mEq/L, physiological uptake is likely to be efficiently inhibited. In addition to the information of FFA levels, evaluation by polar map patterns may facilitate in differentiating abnormal FDG uptake from normal physiological uptake. A diffuse uptake or basal ring-like and/or lateral uptake pattern may be physiological in patients with suspected or known cardiac sarcoidosis, because these patterns were observed in healthy subjects.

In healthy subjects, the cutoff value of FFA0 levels was 0.55 mEq/L as per regression trees. However, there were significant differences in the BMI of the healthy subjects in the HEP and LF groups, and the healthy subjects had a higher BMI than the CS patients. The cutoff value of the FFA0 levels may therefore differ based on population characteristics, such as BMI. When the FFA0 levels are considered, LF is a more effective factor in inhibiting physiological uptake.

One of the main limitations of the present study was that it was a non-randomized study. Because there was a noticeable difference in the inhibition of physiological myocardial ^18^F-FDG uptake between HEP and LF in healthy subjects and in patients with known or suspected CS, we were ethically unable to plan a randomized study. Before having conducted the study, we had supposed that more than 18-h fast must be hard for the study subjects, although we believed longer fast must be effective for inhibiting physiological FDG uptake. We had therefore expected the additional effects of heparin administration on 12-h fast method. However, physiological FDG uptake was not inhibited enough in HEP. After that, we experienced a couple of healthy volunteers with 18-h fast or more who showed FDG uptake was completely inhibited. An 18-h fast was acceptable for them. In addition to this experience, heparin use always takes a risk of heparin-induced thrombocytopenia. Taken together, we planned an 18-h fast study without heparin use. We are uncertain whether additional benefit of heparin use to inhibit physiological FDG uptake overcomes the risk of heparin-induced thrombocytopenia or bleeding. In addition, we did not analyze patients with diabetes mellitus. CS patients often have diabetic complications because of steroid use. Evaluation of ^18^F-FDG PET images in CS patients who use steroids and have diabetes could be important and should thus be addressed in future studies.

The importance of diet followed by extended fasting has been suggested. Cheng et al. found that a 15-h fast with a low-carbohydrate diet had a much lower SUV_max_ of 3.3 SUV (similar to liver and thus approximately 2 on our visual scale) vs. a SUV_max_ of 6.2 in those that had an unrestricted diet and fasted 12 h [24]. Kaneta et al. found that there was no difference in myocardial uptake compared to the length of fasting, but a higher uptake was found in outpatients who presumably have less control over intake than in an inpatient hospital food setting [25]. Probably an optimal protocol for inhibiting physiological uptake may be a combination of all techniques, including a low-carbohydrate and high-fat diet, long fast, and heparin use. Patient's risk and conditions should be considered for the preparation, and long fast (more than 18 h) is recommended in measuring serum free fatty acid levels before scanning.

## Conclusions

The effect of LF on the inhibition of physiological myocardial ^18^F-FDG PET uptake was compared with the effects of HEP in healthy subjects and patients with known or suspected CS. Physiological myocardial uptake was more efficiently inhibited in patients in the LF group compared with in the HEP group. In addition, increased FFA levels may be associated with the inhibition of physiological myocardial uptake. Our data suggest that LF and monitoring FFA0 levels could be helpful in the interpretation of ^18^F-FDG PET images used to evaluate active lesions of CS, resulting in improved diagnosis and management.

## Competing interests

The authors declare that they have no competing interests.

## Authors’ contributions

MiM and MaM participated in the design of the study, carried out the image interpretation, performed the statistical analysis, and drafted the manuscript. KU and JW conducted the FDG PET studies in normal healthy subjects with 18-h fasting before scanning. KK, KI, RM, YM, MO, and OO conducted the FDG PET studies in normal subjects and patients with known or suspected cardiac sarcoidosis who had heparin loading plus 12-h fasting before scanning. TN managed the patients' data on cardiac status. YY and TY managed the patients' data about systematic sarcoidosis. MH conceived of the study and participated in its design and coordination. All authors read and approved the final manuscript.
